# ﻿*Terniopsisyongtaiensis* (Podostemaceae), a new species from South East China based on morphological and genomic data

**DOI:** 10.3897/phytokeys.194.83080

**Published:** 2022-04-18

**Authors:** Miao Zhang, Xiao-Hui Zhang, Chang-Li Ge, Bing-Hua Chen

**Affiliations:** 1 College of Life Sciences, Fujian Normal University, Fuzhou 350117, China Fujian Normal University Fuzhou China; 2 The Public Service Platform for Industrialization Development Technology of Marine Biological Medicine and Products of the State Oceanic Administration, Fujian Key Laboratory of Special Marine Bioresource Sustainable Utilization, Southern Institute of Oceanography, College of Life Sciences, Fujian Normal University, Fuzhou 350117, China Fujian Normal University Fuzhou China

**Keywords:** Biodiversity, chloroplast genome, morphology, phylogeny, taxonomy

## Abstract

The new species *Terniopsisyongtaiensis* X.X. Su, Miao Zhang & Bing-Hua Chen, from Fujian Province, China, is described and illustrated. It is similar to *T.heterostaminata* from Thailand, but differs in its two fertile stamens, fewer but longer vegetative ramuli, fewer but shorter flowering ramuli, shorter pedicels, capsule-stalk and stamens. The complete chroloplast genome of the new species is 129,074 bp long and has a typical quadripartite structure, including two inverted repeat regions (IRs) of 18,504 bp in length, separated by a large single-copy (LSC) and a small single-copy (SSC) regions of 79,000 bp and 13,066 bp, respectively. The *ycf*1 and *ycf*2 genes were lost compared to most higher plants, leading to a substantial reduction in the IR. The phylogenetic analysis using both *matK* and nrITS revealed that *T.yongtaiensis* is sister to *T.heterostaminata* with moderate support, and formed a clade with other *Terniopsis* species, suggesting that the new species belongs to Tristichoideae.

## ﻿Introduction

The Podostemaceae (river-weeds) are unique aquatic angiosperms that exist in various wetlands across the world’s tropics and subtropics (Philbrick and Novelo 1995; Cook 1996; Koi et al. 2015). The plants grow immersed in rapid and turbulent currents and are tightly adhered to the surface of rocks during the rainy season, and then germinate, blossom, produce fruit and finally wither when the water level falls during the dry season. During the rainy season, the seeds are disseminated by wind, birds and running water; the seed coat becomes sticky and adheres to the rock surfaces, and then they germinate and develop seedlings (Tǎng and Kato 2020).

Three subfamilies, Podostemoideae, Weddellinoideae and Tristichoideae are recognized in the family Podostemaceae (Kita and Kato 2001; Koi et al. 2015). Morphologically, Tristichoideae has the least deviation in body plan, with a unique vegetative structure called “ramulus” that arises endogenously in the root tissue and is interpreted as leaf-stem intermediates because they combine typical leaf and typical stem characteristics (Fujinami and Imaichi 2009). There are five genera, viz. *Terniopsis* (= *Malaccotristica*), *Tristicha*, *Indodalzellia*, *Indotristicha*, and *Dalzellia* in the subfamily Tristichoideae (Fujinami and Imaichi 2009; Koi et al. 2009) and only the genus *Terniopsis* is recorded in China (Chao 1948, 1980; Kato and Kita 2003).

Chao proposed *Terniopsissessilis* H.C. Chao, a new genus and species. As name *Terniopsis* with the suffix –*opsis* means a plant similar to *Terniola* (=*Dalzellia*), Chao considered it as allied to *Terniola*. *Terniopsis* was described as a monotypic genus based on its floral traits (solitary or binary, sessile, axillary above the first basal leaves of flowering ramuli, two bracts, and cristate stigma), distinguishing it from Indian *Dalzellia* Wight (Chao 1948). Although the publication of Chao in 1948 was legitimate, it was unfortunately overlooked by authorities, so he redescribed it in 1980 (Chao 1980). Cusset and Cusset believed that the aforementioned characteristics were insufficient to support *Terniopsis* as a new genus, and reduced it under the genus *Dalzellia* Wight, which included *D.carinata* and *D.diversifolia* (Cusset and Cusset 1988). This view was accepted by the FOC (Flora of China) (Qiu and Philbrick 2003). Nevertheless, further molecular phylogenetic studies indicated that *T.sessilis* is sister to *Malaccotristicha* C. Cusset and G. Cusset (1988), and distant from *Dalzelliazeylanica* (type species of *Dalzellia*) (Kita and Kato 2001). Kato and Koi recognized the genus *Terniopsis* (Kato and Kita 2003), which was subsequently revised by Kato to include *Malaccotristicha* and *Dalzellia* sensu Cusset, pro parte, but excluded *D.zeylanica* (type species), as well as recognized *Terniopsismalayana* (=*Malaccotristichamalayana*). Furthermore, Kato included Australian *Tristichaaustralis* in *Terniopsis* as *T.australis* (Kato 2006). There are now 15 species in the *Terniopsis* genus around the world (Kato et al. 2003; Kato 2006; Kato and Koi 2009; Werukamkul et al. 2012; Koi and Kato 2015; Lin et al. 2016), including *T.australis* (C. Cusset & G. Cusset) Kato, *T.brevis* Kato, *T.chanthaburiensis* Kato & Koi, *T.filiformis* Werukamkul, Ampornpan, Koi & Kato, *T.heterostaminata* Werukamkul, Ampornpan, Koi & Kato, *T.malayana* (Dransfield & Whitmore, 1970) Kato, *T.microstigma* Koi & Kato, *T.minor* Kato & Koi, *T.ramosa* Kato, *T.savannaketensis* Koi & Kato, *T.sesadensis* Koi & Kato, *T.sessilis*, *T.ubonensis* Kato, *T.vapyensis* Koi & Kato and *T.daoyinensis* Q.W.Lin, G. Lu & Z.Y.Li.

A *Terniopsis* species that resembles *T.heterostaminata* from Thailand was discovered during our field investigation in Yongtai County, Fujian Province. As a result of comprehensive research, we observed that the species has considerable variation in plant morphology, flower and fruit characteristics, and that its phylogenetic position is supported by molecular-level data. As a result, we conclude that it is a new species, *Terniopsisyongtaiensis*, based on morphological distinctions, geographical isolation, and molecular evidence.

## ﻿Materials and methods

### ﻿Morphological description

The morphological description of the new species was based on the specimens collected in a variety of localities in 2022. A stereoscopic zoom microscope (Carl Zeiss, Axio zoom. v.16, Germany), equipped with an attached digital camera (Axiocam), and a digital caliper were used to record the sizes of the morphological characters. Field observations provided habitats and phenology for the new species.

The leaf sample from Yongtai County, Fujian, China, was collected for DNA extraction.

### ﻿DNA extraction, Genome sequencing, assembly, annotation and analysis

In this study, total DNA was extracted from freeze-dried material using DNeasy Plant Mini Kit (Qiagen, Valencia, CA, USA). Purified total DNA of the new species was fragmented, genome skimming was performed using next-generation sequencing technologies on the Illumina Novaseq 6000 platform with 150 bp paired-end reads and 350 bp insert size by Berry Genomics Co. Ltd. (Beijing, China), and 13.98 GB of reads was obtained.

The paired-end reads were filtered and assembled into complete plastome using GetOrganelle v.1.7.5 with appropriate parameters, with K-merset “21,45,65,85,105” (Jin et al. 2020a). Following previous studies, our workflow includes five key steps as well (Camacho et al. 2009; Bankevich et al. 2012; Langmead and Salzberg 2012; Jin et al. 2020a). Graphs of the final assembly were visualized by Bandage to assess their completeness (Wick et al. 2015). Gene annotation was performed using CPGAVAS2 and PGA. Geneious v.2021.2.2 was used to manually calibrate the start and finish points for disputed positions (Jin et al. 2020a). The different annotations of protein coding sequences were confirmed using BLASTx. The tRNAs were checked with tRNAscan-SE v.2.0.3. Final chloroplast genome maps were created using OGDRAW.

The *matK* sequences were extracted using Geneious v.2021.2.2 from the chloroplast sequences deposited in the GenBank based on the annotated chloroplast genome. The nrDNA (18S-ITS1-5.8S-ITS2-26S) was assembled using GetOrganelle v1.7.5, with –R of 7 and k –merset of“35, 85, 115”, the embplant_nr library was selected as the reference genome database, then annotated and visualized using Geneious v2021.2.2.

### ﻿Phylogenetic analysis

Phylogenetic analyses were conducted using Maximum likelihood (ML) and Bayesian Inference (BI) analyses, based on the *matK* and nrITS sequences. To construct the phylogenetic tree using *matK* sequence, 27 species (Suppl. material 1: Table S1) of *Terniopsis*, *Tristicha*, *Dalzellia*, *Weddellina*, *Polypleurum*, *Zeylanidium* and *Tristellateia* were included in our analysis. A species of *Tristellateia* was selected as outgroup. Each individual sequence was aligned using MAFFT 7.310 (Katoh and Standley 2013) with default settings. A concatenated supermatrix of the two sequences was generated using PhyloSuite v.1.1.15 (Zhang et al. 2019) for the phylogenetic analysis. All missing data were treated as gaps. Gblocks 0.91b (Castresana 2000) was applied to eliminate poorly aligned regions of the concatenated supermatrix with gaps set as no different to other positions. The best nucleotide substitution model according to Bayesian Information Criterion (BIC) was TVM+F+G4, which was selected by Model Finder (Kalyaanamoorthy et al. 2017) implemented in IQTREE v.1.6.8. Maximum likelihood phylogenies were inferred using IQ-TREE (Nguyen et al. 2015) under the model automatically selected by IQ-TREE (‘Auto’ option in IQ-TREE) for 1000 ultrafast (Minh et al. 2013) bootstraps. Bayesian Inference phylogenies were inferred using MrBayes 3.2.6 (Ronquist et al. 2012) under GTR+F+G4 model (2 parallel runs, 2000000 generations), in which the initial 25% of sampled data were discarded as burn-in. Phylograms were visualized in iTOL v.5.

To construct the phylogenetic tree using nrITS, 13 species of *Terniopsis* and *Cladopus* (Suppl. material 1: Table S2) were included. A species of *Cladopus* was employed as outgroup. The study was carried out as described above, and according to the Bayesian Information Criterion (BIC), the optimal nucleotide substitution model was GTR+F+G4.The best nucleotide substitution model according to Bayesian Information Criterion (BIC) was HKY+F+G4, which was selected by Model Finder (Kalyaanamoorthy et al. 2017) implemented in IQTREE v.1.6.8. Maximum likelihood phylogenies were inferred using IQ-TREE (Nguyen et al. 2015) under the model automatically selected by IQ-TREE (‘Auto’ option in IQ-TREE) for 1000 ultrafast (Minh et al. 2013) bootstraps.

## ﻿Results

### ﻿Taxonomic treatment

#### ﻿ Terniopsis yongtaiensis

Taxon classificationPlantaeMalpighialesPodostemaceae

X.X. Su, Miao Zhang & Bing-Hua Chen sp. nov.

BD43D624-B134-55FE-BB92-9B2B62174A1D

urn:lsid:ipni.org:names:77297070-1

Figs 1 , 2 , 3 , 4

##### Type.

China. Fujian: Yongtai County, Fuquan Town, elevation 95 m, 25°51'N, 118°52'E, 2 January 2022, *Bing-Hua Chen* CBH 04587 (Holotype, FNU!, barcode FNU0041314; isotypes FNU!, Barcode FNU0041315).

##### Diagnosis.

*Terniopsisyongtaiensis* is similar to *T.heterostaminata*, a remarkable species from Thailand, by having single flower per flowering ramulus, similar ovary length, same shape of stigma and capsule. However, *T.yongtaiensis* has 2 fertile stamens, less number (1 *vs.* 1–3) but longer (13.0–21.9 mm *vs.*1.4–14 mm) vegetative ramuli, less (1–2 vs. 1–4) but shorter (1.8–5.5 mm *vs.* 1.2–15 mm) flowering ramuli, shorter (1.1–2.5 mm *vs.* 1.7–7 mm) pedicels, shorter (1.9–3.1 mm *vs.* 2.5–8 mm) capsule-stalk, and shorter (1.1–1.3 mm *vs.* 1.5–3 mm) stamens.

The variations in morphology between *T.yongtaiensis* and the other two *Terniopsis* species from China, *T.sessilis* and *T.daoyinensis*, are more obvious. *T.yongtaiensis* shows clear differentiation between vegetative and reproductive stems, the erectness of the ramuli, and the characteristics of flower and fruit are distinctive from those of *T.sessilis* from Changting County, Fujian Province (Table 1, Suppl. material 1: Figs S2–S4). However, *T.daoyinensis* from Hainan differs significantly from other species of the genus by its long (up to 1 mm) and distinctly multi-furcated stigmas (Table 1).

**Table 1.** Comparison of two phylogenetically closely related and two other domestic species of *Terniopsis* from China.

Characteristics	*T.yongtaiensis*	*T.heterostaminata*	*T.sessilis*	*T.daoyinensis*
Root width (mm)	0.3–1.1	0.4–1.6	1–1.5	1–3
Root color	blackish-green	/	purplish- red	/
vegetative ramulus number	1	1–or2–(or3)	1	1
Flowering shoot associated ramulus number	1–2	1–4	1	2–or3
Ramulus length (mm)	1.8–22	1.4–14	7–9	3–30
Flower number per flowering shoot	1	1	1–2	1
Pedicel length (mm)	1.1–2.5	1.7–7	ca. 1.2	4–10
Capsule-stalk length (mm)	1.9–3.1	2.5–8	ca. 1	5–10
Stamen number	2	2 (rarely 3)	2,3	3
Stamen length (mm)	1.1–1.3	1.5–3	0.9–2.5	2–4
Ovary length (mm)	0.9–1.4	0.9–1.5	0.6–0.8	1.5–2
Stigma length (mm)	0.5	0.2–0.5	0.1–0.2	1
Stigma shape	Cristate	Cristate	cristate	multi-furcate
Capsule shape	Obovoid	Obovoid	elliptical	oblong-obovoid
Distribution	China	Thailand, Laos	China	China

##### Description.

**Figure 1.**
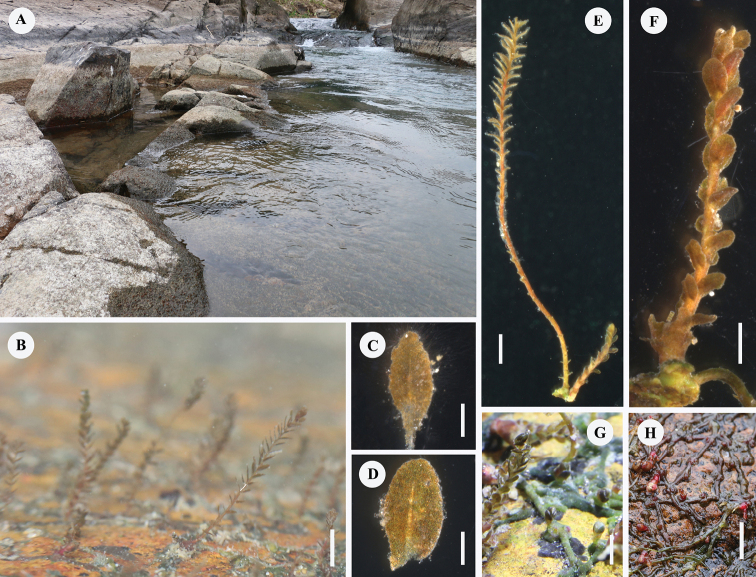
**A** habitat **B** vegetative ramulus, upright, subdistichous (photo in aquarium) **C** leaf on the vegetative ramulus **D** leaf on the fertile ramulus **E** vegetative ramulus (left, long) and fertile ramulus (right,short) **F** fertile ramulus with tristichous leaves **G** flattened ribbon-like roots, (dark green in water) **H** subcylindrical roots (purplish-red at flowering or when water is shallow). Scale bars: 4 mm (**B, H**); 0.4 mm (**C, D**); 2 mm (**E, G**); 0.2 mm (**F**).

**Figure 2.**
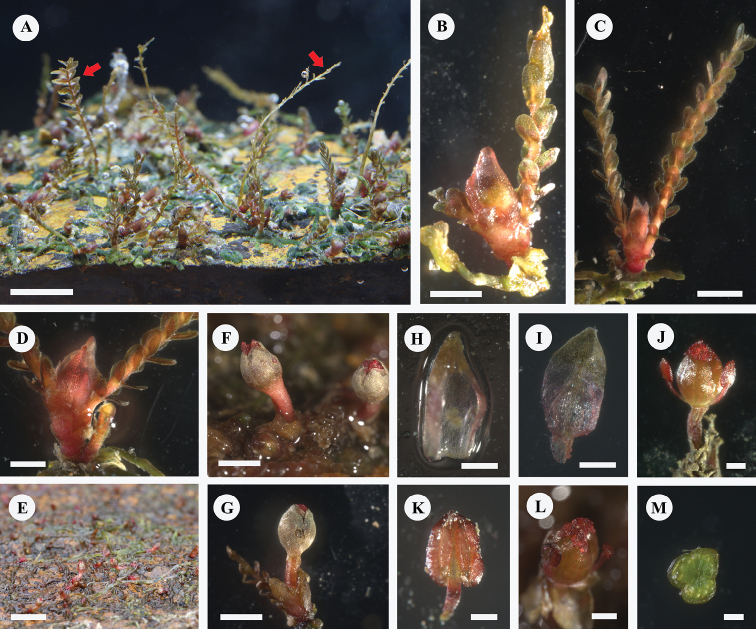
**A** branched flattened root with vegetative ramuli (red arrow) and young flower (shoot) on flank (photo in aquarium) **B, C** flower bud above bracts associated with short shoots (2-ramuli), showing leaves in 3 ranks **D** Young shoot associated with two ramuli and broken vegetative ramulus **E** flowers **F** two flowers at anthesis, showing withered ramuli **G** flower subtended with 2 bracts at base and associated with ramuli, showing pedicel and urceolate corolla **H** bract **I** tepal **J** flower with 2 stamens **K** stamen **L** top oblique view of flower, showing 3 cristate stigmas **M** cross section of the ovary, showing three locules. Scale bars: 5 mm (**A, E**); 1 mm (**B–D, F, G**); 250 μm (**H, I, J, L**); 100 μm (**K**); 200 μm (**M**).

**Figure 3.**
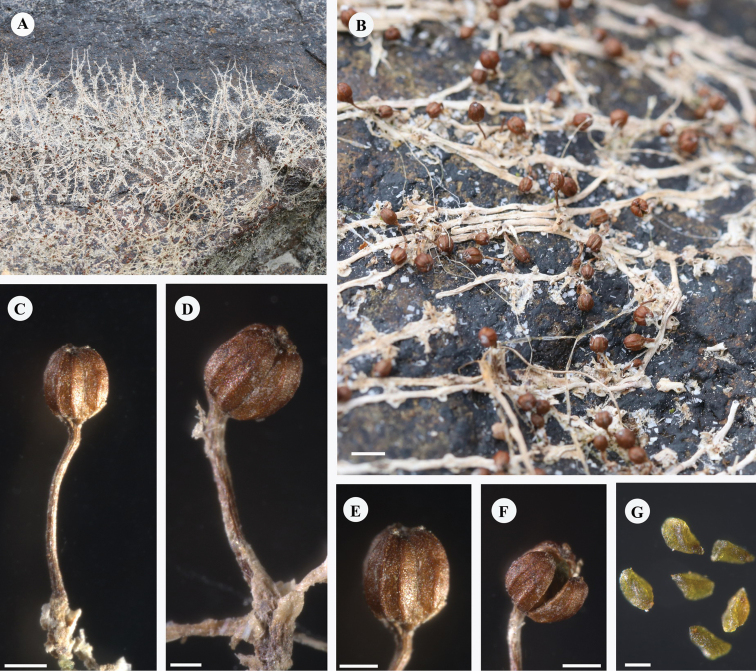
*Terniopsisyongtaiensis***A** plants attached to stone surfaces in patches, withered after fruiting, banded-roots visible, in the dry season when the river level is reduced **B** habitat, showing ripe or nearly ripe fruits and withered roots **C, D** stalked fruit **E** fruit with 9 ribs **F** ripe fruits with dehiscent capsule, showing 3 lobes **G** seeds. Scale bars: 2 mm (**B**); 1 mm (**C**); 0.5 mm (**D, E**); 100 μm (**F**).

Perennial herbs. Ribbon-like roots, flattened to subcylindrical, 0.59 (0.30–1.07) mm wide, 0.58 mm thick, monopodially branched, adhering to rock surface, dark green in water, turns purplish-red or brick-red at flowering or when water is shallow; vegetative ramuli on both flanks of roots, upright, 17.58 (3.00–21.90) mm long, ca. 0.28 mm wide; leaves 48 (39–55), elliptic or spatulate, flattened, sessile, entire, subdistichous; the top leaves are usually larger than the basal ones, 1.73 (0.96–1.66) mm long, 0.65 (0.56–0.76) mm wide, the basal leaves gradually fall off during growth; flowering shoots grow lateral to vegetative ramuli, with a single flower and 1–2 associated upright ramuli, 3.14 (1.76–5.53) mm long, 0.31 mm wide, each has 24 (17–32) leaves, 0.93 (0.61–1.24) mm long, 0.53 (0.35–0.75) wide, elliptic or broad-ovate, tristichous, subequal, smaller than leaves on vegetative remuli (Fig. 1), all ramuli and leaves wither when fruiting. Flowers bisexual, small, solitary, petiolate, grows in axils of first leaves at base of flowering shoots; bracts 2, helmet-shaped, thinly membranous, pink or light red, 1.27 (1.08–1.61) mm long, 1.09 (0.80–1.45) mm wide; pedicel, 1.58 (1.13–2.52) mm long, ca. 0.41 mm in diameter; tepals, ca. 1.05 mm long, ca. 1.12 mm wide, shallowly lobed, lobes 3, red purple, semicircular, ca. 0.42 mm long, ca. 0.68 mm wide, lower part of tepals unite urceolated, turns to white when flowering; stamens 2, 1.21 (1.14–1.33) mm long, with introrse anthers, less than the perianth lobes, short filaments, segregate, base attached to ovary, 0.59 mm long; anthers 4, elliptic, 0.61 mm long, endocentric, rounded at the base. Ovaries elliptic, 3-locular, 1.13 (0.94–1.39) mm long, 1.03 (0.90–1.22) mm wide; ovules, 34 per locule; stigmas 3, padded, cristate, 0.16 mm tall, 0.49 mm long, 0.43 mm wide (Fig. 2). Capsule, 9-ribbed, obovoid, 1.15 (1.01–1.52) mm long, 0.98 (0.78–1.25) mm wide, fissured into 3 equal pieces at maturity; Capsules stalked, 2.48 (1.87–3.07) mm long; seeds ca. 25, green, teardrop-shaped, slightly concave at top, 0.21 (0.19– 0.24) mm long (Fig. 3).

Florescence December to January, fruiting season January to February.

##### Distribution, habitat and conservation status.

*Terniopsisyongtaiensis* is only known from Yongtai, Fujian, China (Suppl. material 1: Fig. S1), where it grows on rocks in unpolluted streams, sometimes covering the entire surface of the rock. Many other plants grow in the surrounding habitat, whose tree layer includes *Ficusmicrocarpa* L. f. (Moraceae), *Prunusmume* Sieb. (Rosaceae), *Rhuschinensis* Mill. Anacardiaceae, *Scheffleraheptaphylla* (Linnaeus) Frodin (Araliaceae) and others;the shrub layer includes *Ficuserecta* Thunb. (Moraceae), *Callicarpakochiana* Makino (Lamiaceae), *Buddlejaasiatica* Lour. (Scrophulariaceae), *Adina rubella* Hance (Rubiaceae) and others; the vegetation layer includes *Polygonumlapathifolium* L. (Polygonaceae), *P.chinense* L. (Polygonaceae), *Rubushirsutus* Thunb.(Rosaceae), *Ludwigiaepilobioides* Maxim.(Onagraceae), *Colocasiaantiquorum* Schott (Araceae), *Panicumrepens* L. (Poaceae), *Miscanthusfloridulus* (Lab.) Warb. ex Schum et Laut. (Poaceae), *Neyraudiareynaudiana* (kunth.) Keng (Poaceae), *Isachneglobosa* (Thunb.) Kuntze (Poaceae), *Saccharumarundinaceum* Retz. (Poaceae), *Commelinacommunis* L. (Commelinaceae), *Musanana* Lour. (Musaceae) and others; the interlayer plants includes *Cocculusorbiculatus* (L.) DC. (Menispermaceae), *Puerariamontana* (Loureiro) Merrill (Fabaceae) and others; and some exotic plants includes *Alternantheraphiloxeroides* (Mart.) Griseb. (Amaranthaceae), *Myriophyllumaquaticum* (Vell.) Verdc. (Haloragaceae), *Bidenspilosa* L. (Asteraceae) and others.

*Conservation status*: According to our investigation, *Terniopsisyongtaiensis* was only found in a stream in Yongtai County, Fujian Province, China and hence, we suggest its placement in the Data Deficient category of IUCN (2022). In addition, according to the Updated List of National Key Protected Wild Plants (Decree No. 15) by the country’s State Forestry and Grassland Administration and the Ministry of Agriculture and Rural Affairs, all of the known genera of Podostemaceae found in China are classified as under national secondary protection. This new species should also be included on the national secondary protection list during the upcoming revision process.

**Table 2.** Gene contents in the plastid genome of *Terniopsisyongtaiensis*.

Category, Group of Genes	Gene Names
**Photosynthesis**:	
Subunits of ATP synthase	*atpA*, *atpB*, *atpE*, atpF*, *atpH*, *atpI*
Subunits of NADH dehydrogenase	ndhA*, ndhB*(x2), *ndhC*, *ndhD*, *ndhE*, *ndhF*, *ndhG*, *ndhH*, *ndhI*, *ndhJ*, *ndhK*
Cytochrome b/f complex	*petA*, *pet*B*, petD*, *petG*, *petL*, *petN*
Subunits of photosystem I	*psaA*, *psaB*, *psaC*, *psaI*, *psaJ*
Subunits of photosystem II	psbA, psbB, psbC, psbD, psbE, psbF, psbH, psbI, psbK, *psbJ*, *psbL*, *psbM*, *psbN*, *psbT*
Large subunit of rubisco	*rbcL*
**Other genes**:	
Subunit of Acetyl-CoA-carboxylase	*accD*
c-type cytochrome synthesis gene	*ccsA*
Envelope membrane protein	*cemA*
Maturase	*matK*
**Self-replication**:	
Large subunit of ribosome	rpl2*(x2), *rpl14*, rpl16*, *rpl20*, *rpl23* (x2), *rpl33*, *rpl36*
DNA dependent RNA polymerase	*rpoA*, *rpoB*, rpoC1*, *rpoC2*
Small subunit of ribosome	*rps2*, *rps3*, *rps4*, *rps7* (x2), *rps8*, *rps11*, rps12*^a^ (x2), *rps14*, *rps 15*, *rps18*, *rps19*
rRNA Genes	*rrn4.5S* (x2), *rrn5S* (x2), *rrn16S* (x2), rrn23S*(x2)
tRNA Genes	trnA-UGC*(x2), *trnC-GCA*, *trnD-GUC*, *trnE-UU*C, *trnF-GAA*, *trnfM-CAU*, *trnG-GCC*, *trnH-GUG*, trnI-GAU*(x2), *trnI-CAU* (x2), trnK-UUU*, *trnL-CAA* (x2), trnL-UAA*, *trnL-UAG*, *trnM-CAU*, *trnN-GUU* (x2), *trnP-UGG*, *trnQ-UUG*, *trnR-ACG* (x2),*trnR-UCU*,trnS-UGA*,*trnS-GC*U,*trnS-GGA*, *trnT-CGU*, *trnT-GGU*, *trnT-UGU*, *trnV-GAC* (x2), trnV-UAC*, *trn W-CCA*, *trnY-GUA*
**Unknown function**:	
Conserved open reading frames	*ycf*3*,*ycf*4, *inf*A

Note:* genes containing introns; (x2) genes present as two copies in the IR regions;^a^ indicates trans-spliced gene.

**Figure 4.**
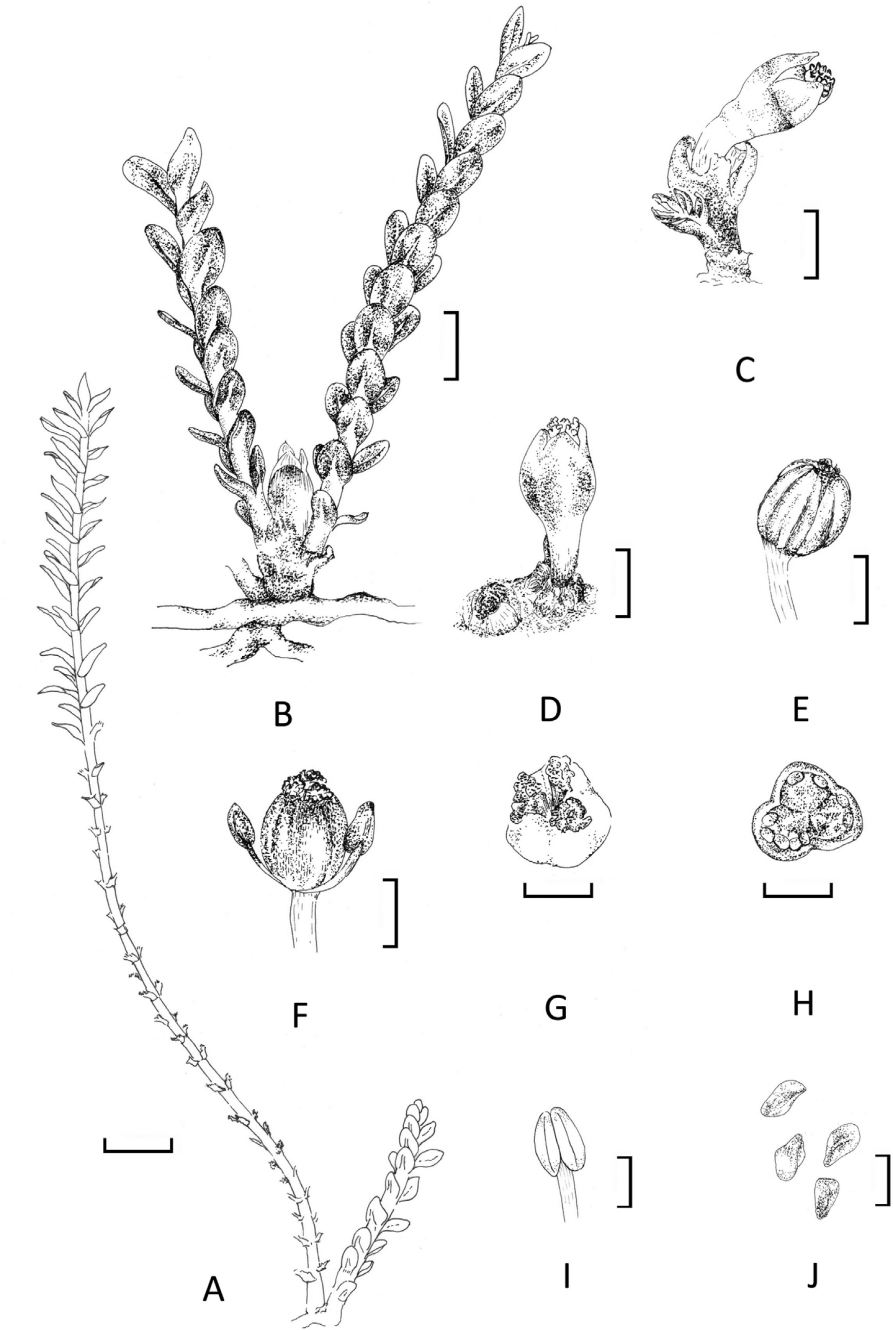
Illustration of *Terniopsisyongtaiensis***A** vegetative ramulus (left, long) and fertile ramulus (right, short) **B** flower bud above bracts associated with short shoots (2-ramuli) **C** flower subtended with 2 bracts at base and associated with ramulus **D** flower at anthesis, showing withered ramuli **E** fruit with 9 ribs **F** flower with urceolate corolla removed, 2 stamens on side of ovary **G** cristate stigmas **H** cross section of the ovary **I** stamen **J** seeds. Scales bars: 1 mm (**A**); 500 μm (**B**); 250 μm (**C–H**); 100 μm (**I**); 50 μm (**J**).

##### Etymology.

The epithet *yongtaiensis* (永泰) refers to Yongtai County, Fujian Province where this new species was found.

### ﻿Characteristics of the *Terniopsisyongtaiensis* plastome

**Figure 5.**
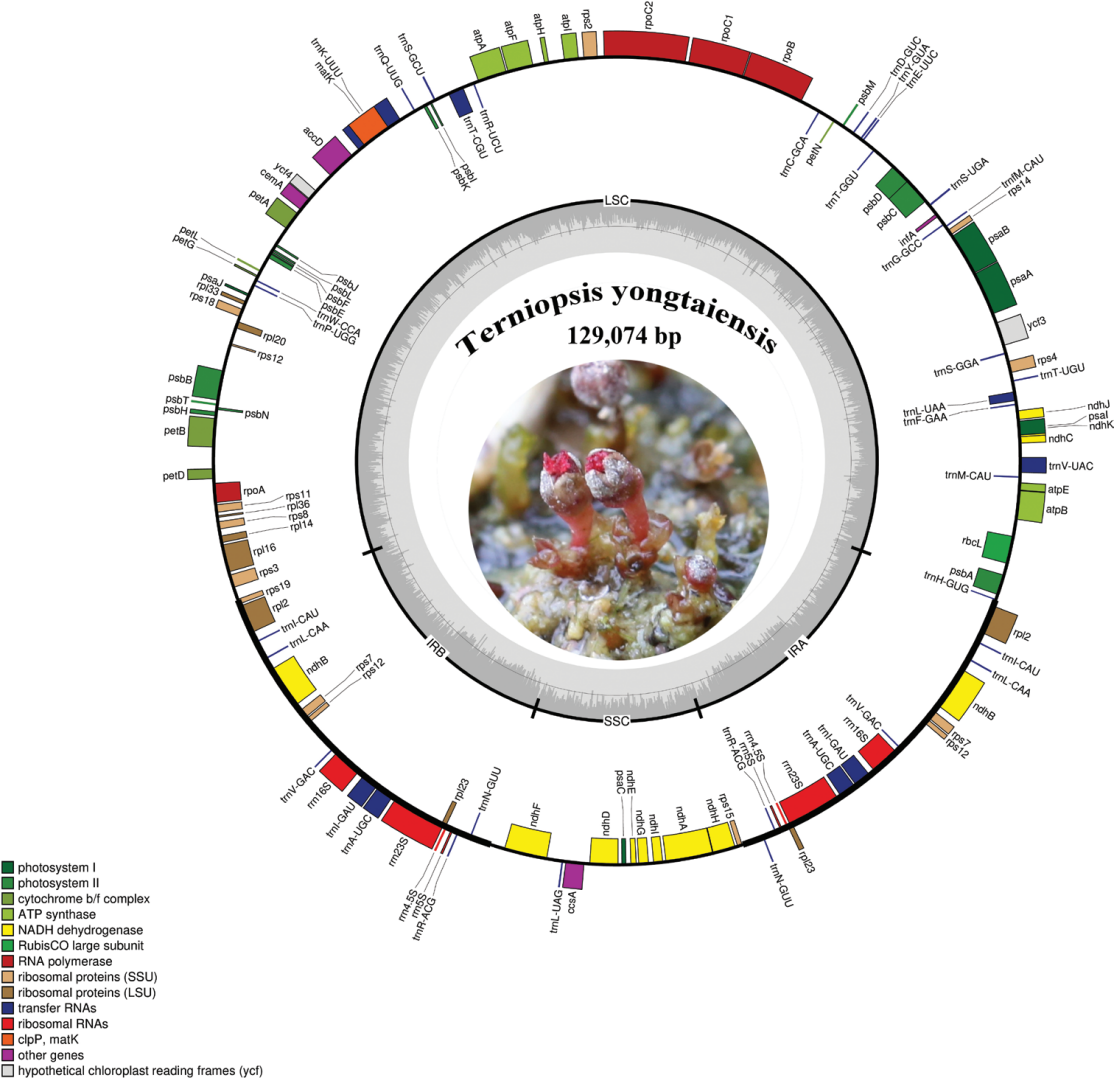
Circular gene map of the plastid genome of *Terniopsisyongtaiensis.* Genes inside the circle are transcribed clockwise, while those drawn outside are transcribed counterclockwise. Genes are color-coded according to their functional groups. The circle inside the GC content graph marks the 50% threshold.

The plastome of *Terniopsisyongtaiensis* (Fig. 5) is 129,074 bp in length, and exhibits a typical quadripartite structure, consisting of a large single copy (LCS) region of 79,000 bp and a small single copy (SSC) region of 13,066 bp, which were separated by a pair of 18,504 bp inverted repeat regions (IRs). The gene map of *T.yongtaiensis* is presented in Fig. 5. The gene composition in plastome of *T.yongtaiensis* would be divided into four categories: gene related to photosynthesis, genes related to self-replication, protein-coding genes with unknown functions, and other genes. A total of 106 unique genes were identified in the plastome; it contains 72 protein-coding genes, 30 tRNAs, and 4rRNAs. A total of 16 genes were duplicated in the IR regions, including *ndhB*, *rpl2*, *rp*l23, *rps*7,*rps*12, *rrn*4.5*S*, *rrn*5*S*, *rrn*16*S*, *rrn23S*, *trnA*-*UGC*, *trnl*-*GAU*, *trnl*-*CAU*, *trnL*-*CAA*, *trnN*-*GUU*, *trnR*-*ACG*, *trnV*-*GAC*. A total of six genes were lost, including *psbZ*, *clpP*, *rpl* 22, *rpl*32, and uncommon losses of *ycf1* and *ycf2*. The annotated plastome was documented in GenBank (accession number OM717943).

### ﻿Comparative analysis of the plastomes

A comparison of the plastome of *Terniopsisyongtaiensis* is made to five other species of Podostemaceae with available data (Table 3). The plastome lengths of the six species varied from 129,074 bp (*T.yongtaiensis*) to 134,912 bp (*Apinagiariedelii*), with *T.yongtaiensis* being the shortest. For the LCS and SSC regions, the extent of length variation between these species is not evident. The number of PCGs in these species is similar to that of most angiosperms, according to a comparative analysis of gene content (Jin et al. 2020b). The numbers of tRNA and rRNA genes, as well as the GC content, are substantially conserved in all of these plastomes, as shown by our findings. In all compared species, the *ycf*1 and *ycf*2 genes, which are two giant open reading frames found in most higher plants, are lost. In *T.yongtaiensis* and *Tristichatrifaria*, the *rps*15 *gene* is found at the SSC/IR border, but it is shifted to IRs in *Apinagiariedelii*, *Marathrumutile*, *M.capillaceum* and *M.foeniculaceum* due to the expansion at the IR/SSC boundary. In *T.yongtaiensis*, the *trnG*-*UCC* gene mutates to *trnT*-*CGU*, and in *M.capillaceum*, it is lost. Further, all the compared species have a gene inversion from *trnK*-*UUU* to *rbcL* in the LSC region, and the gene inversions are of similar size (ranging from 50.4 kb for *T.yongtaiensis* to 52 kb for *A.riedelii*). It represents an essential mechanism for plastome rearrangements (Mower and Vickrey 2018).

**Table 3.** Statistics on the basic features of the plastid genomes of *Terniopsisyongtaiensis* and related taxa.

Species	Voucher	Accession no.	Length (bp)	LSC (bp)	SSC (bp)	IR(bp)	GC content (%)	No. of PCGs	No. of tRNA	No. of rRNA
*Terniopsisyongtaiensis*	CBH 04587	OM717943	129,074	79,000 (~61.2%)	13,066 (~10.1%)	18,504 × 2 (~28.7%)	36.20	72	30	4
*Apinagiariedelii*	C.P. Bove 2513 (R)	MN165812	134,912	85,377 (~61.0%)	12,437 (~8.9%)	21,049 × 2 (~30.1%	34.90	74	30	4
*Marathrumutile*	AMB 497 (ANDES)	MN165814	131,951	79,778 (~60.5%)	12,283 (~9.3%)	19,945 × 2 (~30.2%)	35.10	73	29	4
*Marathrumcapillaceum*	C.P. Bove 2493 (R)	MN165813	134,374	79,990 (~59.5%)	12,302 (~9.2%)	21,041 × 2 (~31.3%)	35.00	75	30	4
*Marathrumfoeniculaceum*	W. D. Stevens – 32072	MK995178	131,600	79,506 (~60.4%)	12,262 (~9.3%)	19,916×2 (~30.3%)	35.10	76	30	4
*Tristichatrifaria*	A. Mesterhazy MLI 128(Z)	MN165816	130,285	78,925 (~60.6%)	12,662 (~9.7%)	19,349 × 2 (~29.7%)	36.40	74	30	4

### ﻿Phylogenetic analysis

**Figure 6.**
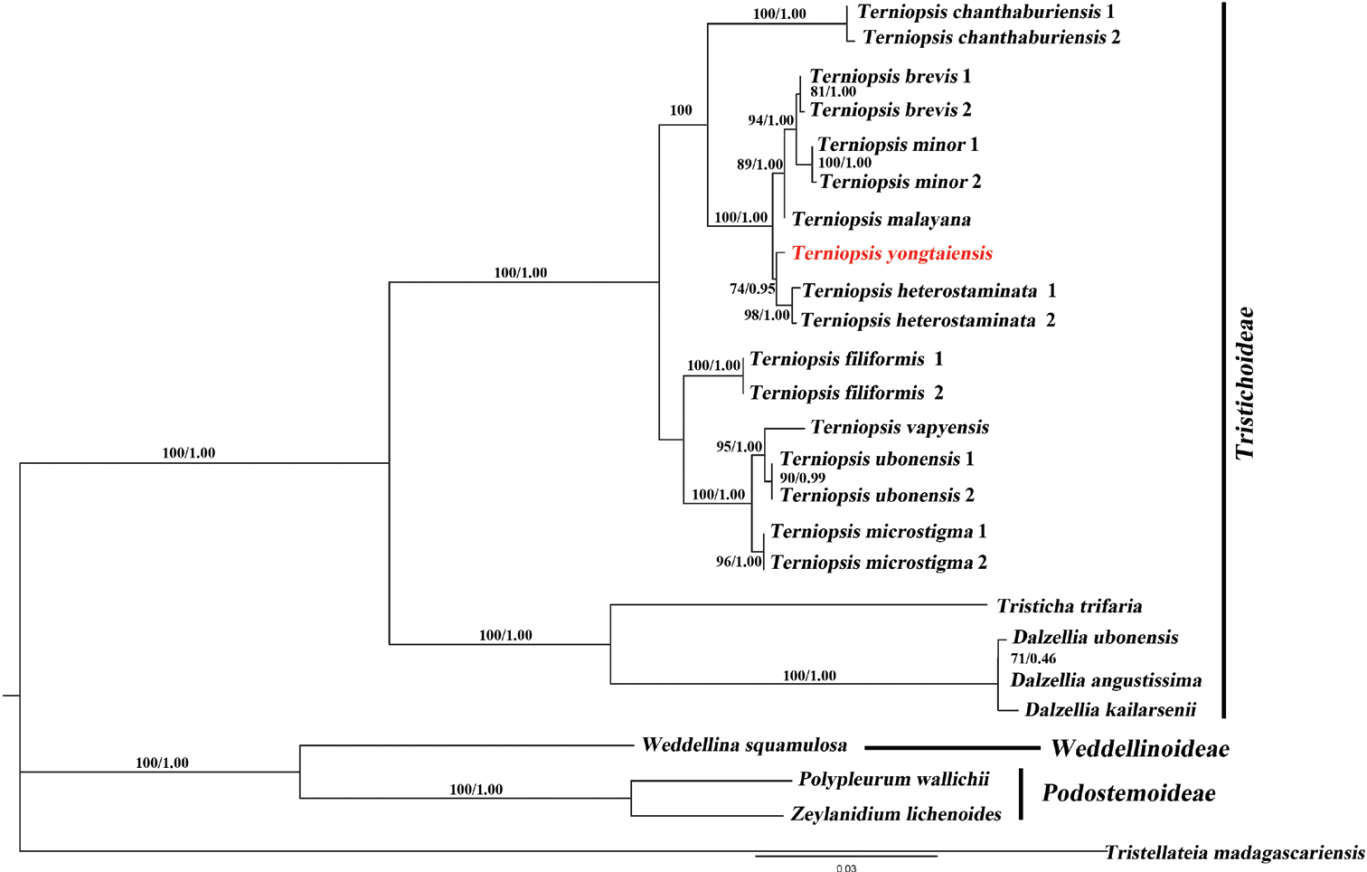
Phylogenetic tree of Asian Podostemaceae based on Bayesian Inference of *matK* sequences. Numbers above and below branches indicate RAxML (left) bootstrap probabilities (BP) and Bayesian (right) posterior probabilities (PP), respectively.

Phylogenies were reconstructed by Maximum likelihood (ML) and Bayesian Inference (BI) analyses using the *matK* and nrITS sequences. The phylogenetic analysis based on *matK* sequences suggested that *Terniopsisyongtaiensis* is sister to *T.heterostaminata* with moderate support, and nested in a clade formed by *T.brevis*, *T.minor*, *T.malayana* with strong support (Fig. 6). Similar results showed by the phylogenetic analysis based on nrITS, suggested *T.yongtaiensis* is closely related to *T.heterostaminata* with moderate support, and sister to a clade comprising *T.chanthaburiensis*, *T.filiformis*, *T.vapyensis*, *T.microstigma*, *T.ubonensis*, *T.savannaketensis*, and *T.malayana* (Suppl. material 1: Fig. S5).

## ﻿Discussion

The *Terniopsissessilis* Chao was first discovered in 1948 in the Tingjiang River basin of Changting County in northwest Fujian Province (Chao 1948, 1980). The literatures indicated that this species has a wide distribution, but to date, 80 years after its report, it has not been found elsewhere after a long and continuous investigation, such as around the Min River, under the Wanshou Bridge (i.e. Jiefang Bridge) in Cangshan District, Fuzhou City, Fujian Province, where a distribution has been noted. This is possibly due to environmental changes and urbanization. Fortunately, some botanical enthusiasts discovered plants that were morphologically similar in Guilin, Guangxi Zhuang Autonomous Region, which our team analyzed and determined were consistent with *T.sessilis* based on *matK* sequences (data not published).

While looking for other distribution sites of *T.sessils* in Fujian Province, the new species *T.yongtaiensis* was discovered in Yongtai county; it differs greatly in appearance from *T.sessilis* (Suppl. material 1: Figs S2–S4), especially in the ramuli, flower and fruit. Roots of *T.yongtaiensis* are often dark green in water, and the vegetative and flowering ramuli can be clearly distinguished. There are more leaves on vegetative ramuli (up to 55), the leaves are spatulate, and they wither during flowering. The ramuli of *T.sessilis*, on the other hand, are often attached to rock surfaces, and are obviously shorter (7–9 mm long), and have fewer leaves (< 12). The number of flowering ramuli branches varies between *Terniopsis* species. The flowering ramuli of *T.yongtaiensis* are usually two-branched, with one flower. The flowering ramuli are shorter and single branched with one or two flowers, but the leaf shape is similar. And the flowering ramuli of *T.heterostaminata* are often single to four-branched, with one flower (Chao 1980; Fujinami and Imaichi 2009; Koi and Kato 2015)

The plastome of *T.yongtaiensis* was compared with the plastome of 5 other species within the Podostemaceae family. All of the studied species lack the *ycf*1 and *ycf*2 genes, which are giant open reading frames found in most higher plants, resulting in a significant reduction of IR regions, thus reducing the size of their plastomes. Based on the available data, we believe that the absence of *ycf*1 and *ycf* 2 genes is typical for Podostemaceae. The *ycf*1 and *ycf*2 genes were also lost in the plastome of Poaceae (Guisinger et al. 2010), Geraniaceae (Weng et al. 2014) and Ericaceae (Braukmann et al. 2017). There is still debate over the functions of the *ycf*1 and *ycf*2 genes, and they have yet to be classified as genes involved in genetic or photosynthetic systems (Drescher et al. 2000).

According to molecular data on *matK* comparison, the new species from Yongtai was closely related to *T.heterostaminata* from Thailand, and was in the sister group of the same cluster in the phylogenetic tree. Additionally, due to its geographical distance and the unique river habitat, this species was identified as a new species and named *T.yongtaiensis*. Investigations of other rivers in Yongtai and surrounding counties have revealed that the species was only found in the upper reaches of the first discovery site, indicating that the species has a very limited distribution area. Meanwhile, a whole-genome analysis will be carried out to ascertain its phylogenetic and evolutional position among angiosperms.

## ﻿Conclusion

*Terniopsisyongtaiensis* should be classified as a new species of Tristichoideae, based on the facts presented in the current study. The plastome of species of genus *Terniopsis* was studied for the first time, and the discovery of *T.yongtaiensis* provides new supporting materials for the phylogeny and evolution for the Podostemaceae family.

### ﻿Key to the species of *Terniopsis* H. C. Chao

**Table:** 

1	Stamens at least two times longer than ovary	**2**
–	Stamens as long as ovary	**5**
2	Stamens 3; stigmas up to 1mm, distinctly multi-furcate	**4. *T.daoyinensis***
–	Stamens 2 or 3; stigmas less than 0.5mm, cristate	**3**
3	Ramulus 10–90mm long; stamen 5–6mm long	**14. *T.ubonensis***
–	Ramulus <5mm long; stamen <5mm long	**4**
4	Stamens 2, 2.5 times as long as ovary	**11. *T.savannaketensis***
–	Stamens 2 or 3, 2 times as long as ovary	**15. *T.vapyensis***
5	Stigmas ≤ 0.2 mm long	**6**
–	Stigmas more than 0.2 mm long	**10**
6	Stigmas simple to laciniate; pedicel 10–15 mm; capsule-stalk 15 mm	**1. *T.australis***
–	Stigmas cristate; pedicel < 1mm; capsule-stalk <10 mm	**7**
7	Pedicel ca. 0.5, ramulus 2–5	**8. *T.microstigma***
–	Pedicel >1mm, ramulus 1–4	**8**
8	Root 2 mm wide; shoot to 30mm long, many times branched; bracts several	**10. *T.ramosa***
–	Root <2 mm wide; shoot to 10mm long, bracts 2	**9**
9	Ramulus <10 mm long; ovary 0.6–0.8 mm; capsula elliptical	**13. *T.sessilis***
–	Ramulus up to 30mm long; ovary 1.5–2.0 mm; capsula obovate	**7. *T.matayana***
10	Stamens 3, rarely 2; stigmas forked, filiform at maturity	**5. *T.filiformis***
–	Stamens 2; stigmas cristate	**11**
11	Vegetative ramuli up to14 mm long	**12**
–	Vegetative ramuli less than 10 mm long	**14**
12	Pedicel 3–14 mm long	**3. *T.chanthaburiensis***
–	Pedicel < 3 mm long	**13**
13	Ramuli associated with flowers 2–4, 2–6 mm long	**6. *T.heterostaminata***
–	Ramulus associated with flowers 1, to 2 mm long	**16. *T.yongtaiensis***
14	Ramuli associated with flowers 4–7 mm long	**9. *T.minor***
–	Ramuli associated with flowers 2–4 mm long	**15**
15	Pedicel 1.3–1.8 mm, ovary 1.3–1.5 × 0.8 mm	**12. *T.sesadensis***
–	Pedicel 3 mm, ovary 0.8–1.3 × 0. 5 mm	**2. *T.brevis***

## Supplementary Material

XML Treatment for Terniopsis yongtaiensis

## Supplementary materials

Supplementary material 1 Appendix Data type doc file Explanation note **Fig. S1.** Distribution of *Terniopsisyongtaiensis*, *T.sessilis* and *T.daoyinensis* of genus *Terniopsis* from China. Legend ▲*T.yongtaiensis*, ◆*T.sessilis*, ●*T.daoyinensis*. **Fig. S2.** Habit and habitat of *Terniopsissessilis*. **Fig. S3.***Terniopsissessilis*, showing stems (ramuli) arising laterally from root, distichous and leaves borne on ramuli in 3 ranks. **Fig. S4.***Terniopsissessilis*, showing two flower buds axillary to the basal leaf, sessile. **Fig. S5.** Phylogenetic tree of Asian Podostemaceae based on Bayesian Inference of nrITS sequences. Numbers above and below branches indicate RAxML (left) bootstrap probabilities (BP) and Bayesian (right) posterior probabilities (PP), respectively. **Table S1.** List of taxa from Podostemaceae and NCBI accession numbers (*matK*). **Table S2.** List of taxa from Podostemaceae and NCBI accession numbers (*nrITS*). https://binary.pensoft.net/file/673911This dataset is made available under the Open Database License (http://opendatacommons.org/licenses/odbl/1.0/). The Open Database License (ODbL) is a license agreement intended to allow users to freely share, modify, and use this Dataset while maintaining this same freedom for others, provided that the original source and author(s) are credited.Miao Zhang, Xiao-Hui Zhang, Chang-Li Ge, Bing-Hua Chen
