# Supplementary material for: ﻿Terniopsisyongtaiensis (Podostemaceae), a new species from South East China based on morphological and genomic data
Source: PhytoKeys. 2022 Apr 18;194:105–22. doi: 10.3897/phytokeys.194.83080 (PMC9038898; doi:10.3897/phytokeys.194.83080)
Supplement: Supplementary material 1 — Appendix [file phytokeys-194-105-s001.doc]

Appendix


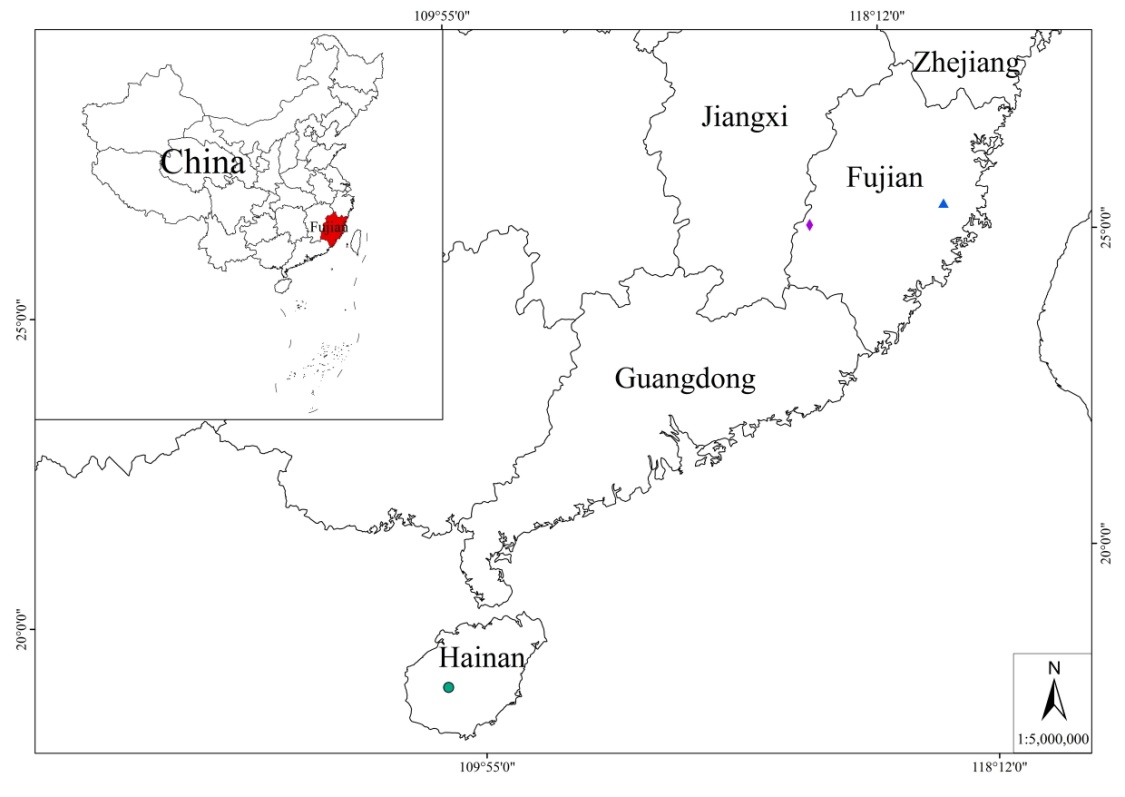


**Figure S1** Distribution of *Terniopsis yongtaiensis*, *T. sessilis* and *T. daoyinensis* of genus *Terniopsis* from China. Legend ▲*T. yongtaiensis*, ◆*T. sessilis*, ●*T. daoyinensis*


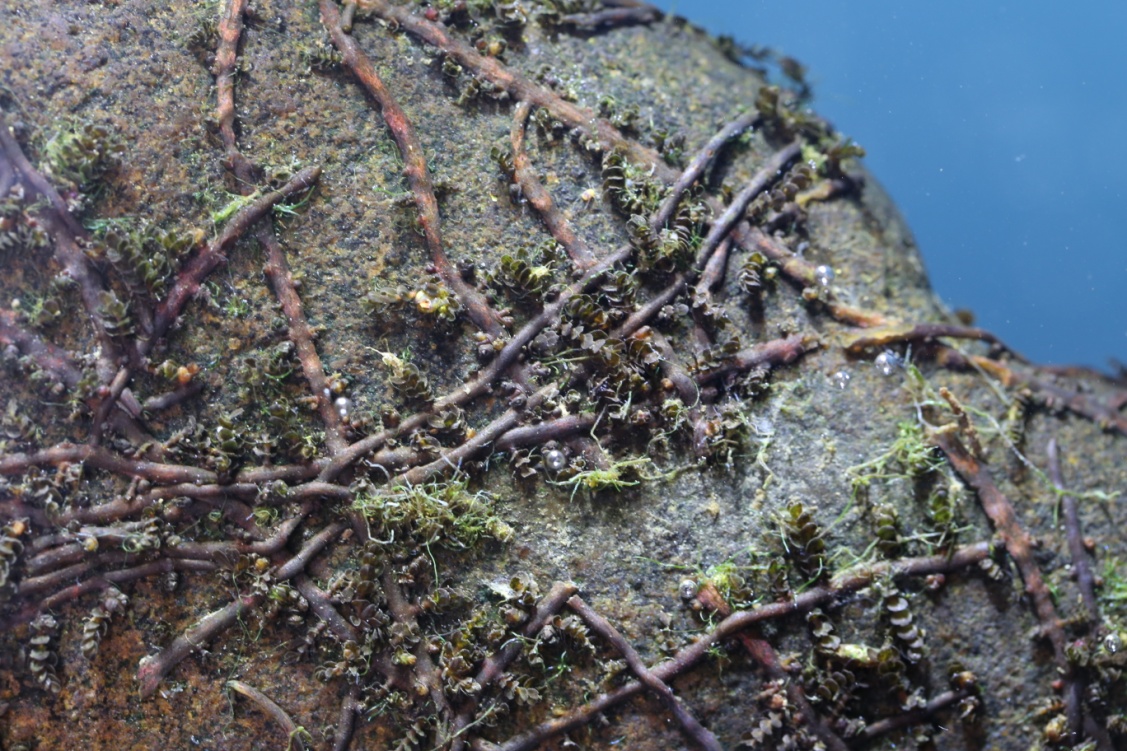


**Figure S2** Habit and habitat of *Terniopsis sessilis*


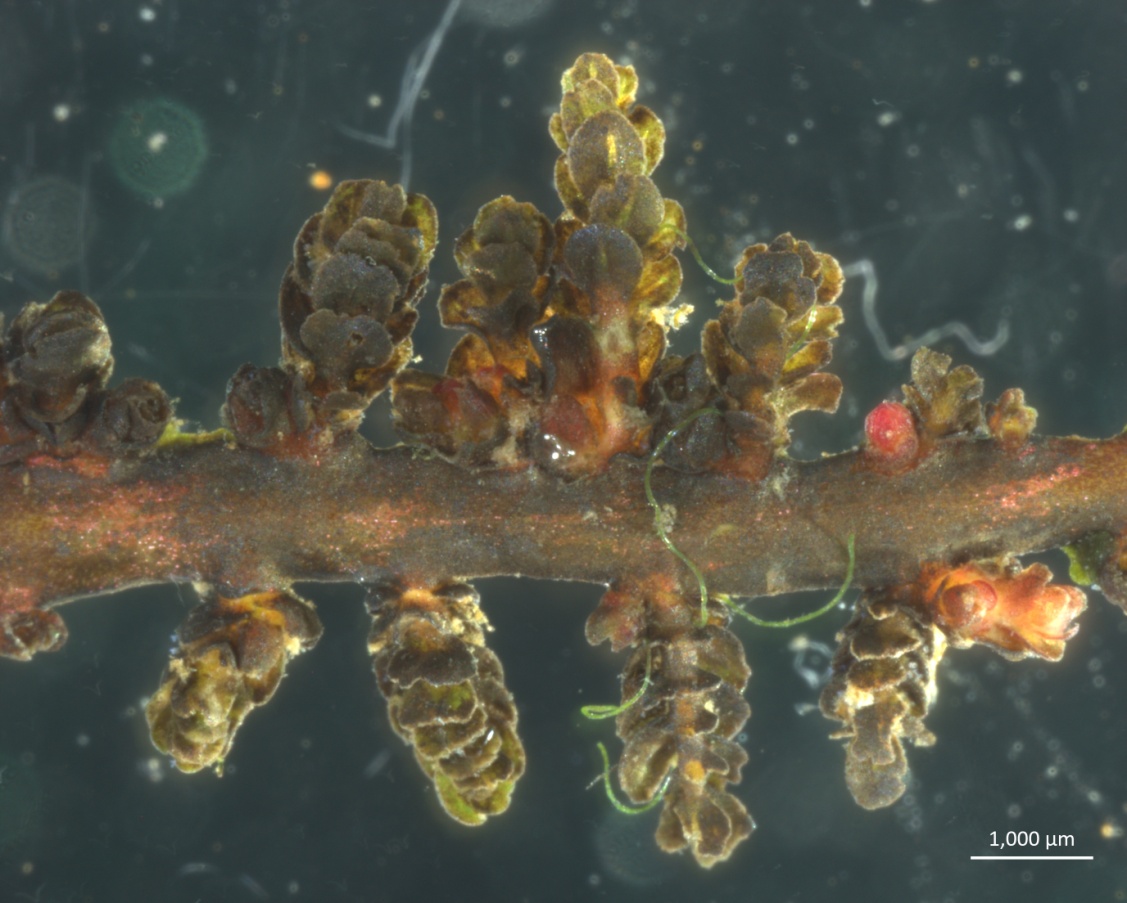


**Figure S3** *Terniopsis sessilis*, showing stems (ramuli) arising laterally from root, distichous and leaves borne on ramuli in 3 ranks.

*
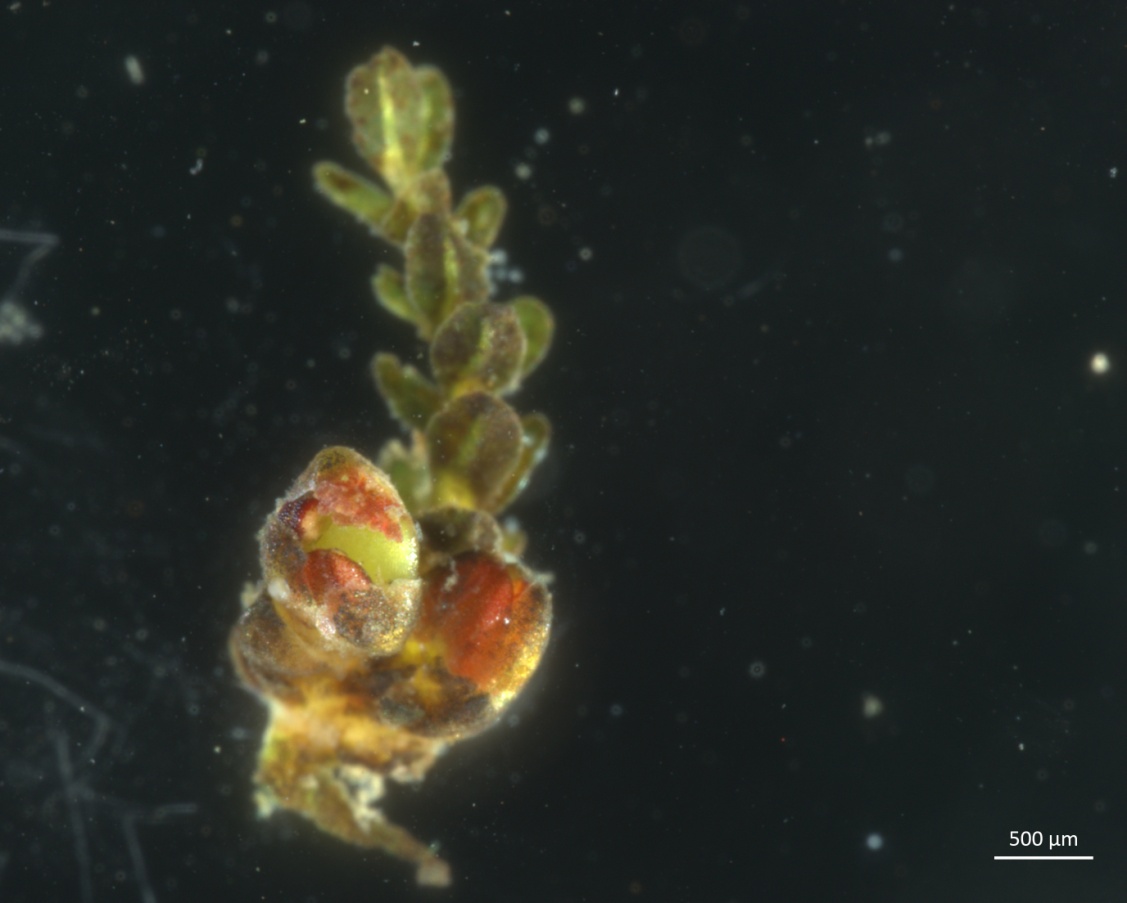
*

**Figure S4** *Terniopsis sessilis*, showing two flower buds axillary to the basal leaf, sessile.

**
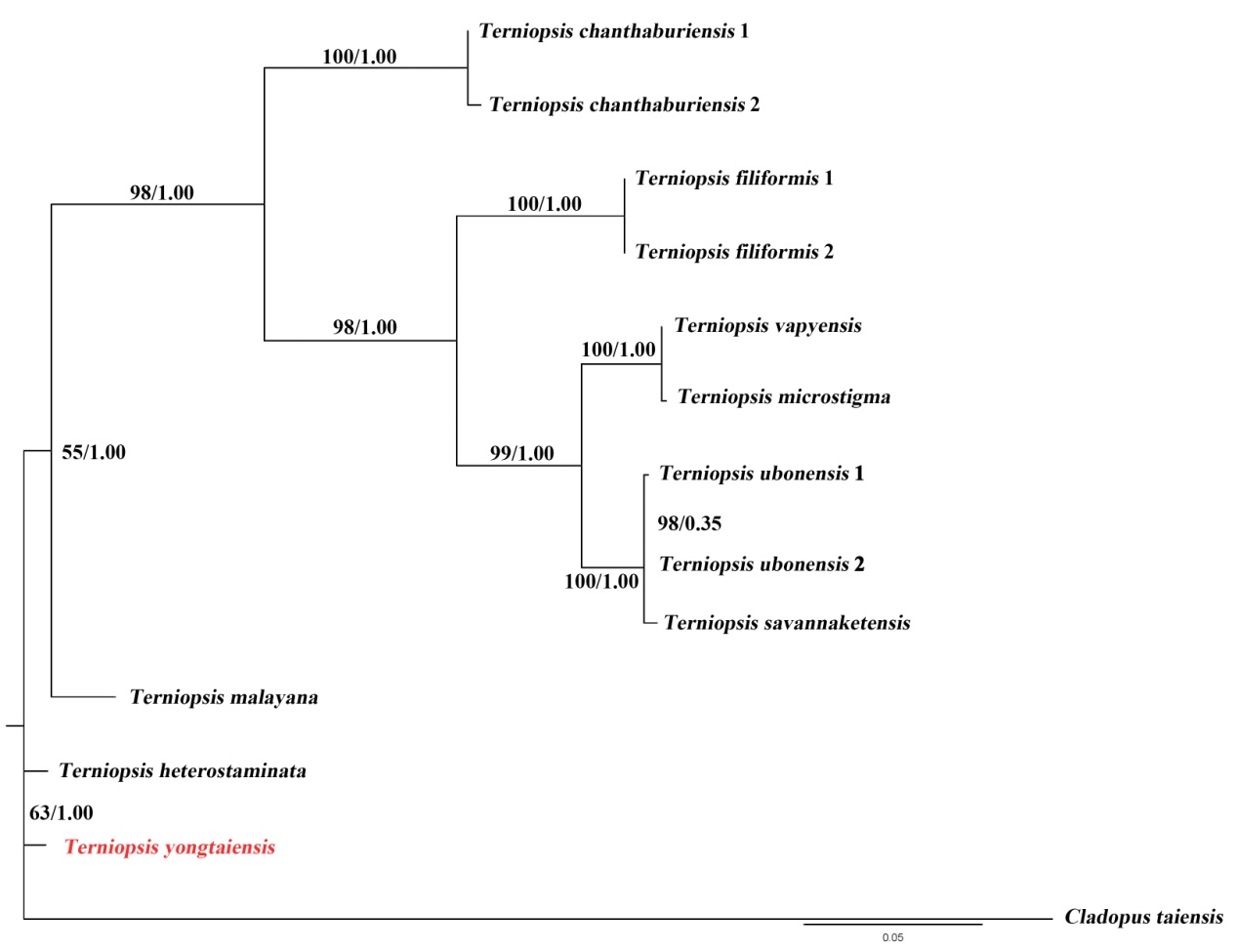
**

**Figure A5** Phylogenetic tree of Asian Podostemaceae based on Bayesian Inference of nrITS sequences. Numbers above and below branches indicate RAxML (left) bootstrap probabilities (BP) and Bayesian (right) posterior probabilities (PP), respectively.

**Table S1 List of taxa from Podostemaceae and NCBI accession numbers (*matK*)**

| 1. No. | Taxa | NCBI accession no. |
| --- | --- | --- |
| 1 | *Terniopsis yongtaiensis* | OM953517.1 |
| 2 | *Terniopsis chanthaburiensis 1* | LC625800.1 |
| 3 | *Terniopsis chanthaburiensis 2* | LC384161.1 |
| 4 | *Terniopsis brevis 1* | LC342961.1 |
| 5 | *Terniopsis brevis 2* | LC342884.1 |
| 6 | *Terniopsis heterostaminata 1* | LC342918.1 |
| 7 | *Terniopsis heterostaminata 2* | LC342903.1 |
| 8 | *Terniopsis filiformis 1* | LC384159.1 |
| 9 | *Terniopsis filiformis 2* | LC384156.1 |
| 10 | *Terniopsis vapyensis* | LC342923.1 |
| 11 | *Terniopsis minor 1* | LC342893.1 |
| 12 | *Terniopsis minor 2* | LC342891.1 |
| 13 | *Terniopsis ubonensis 1* | AB450500.1 |
| 14 | *Terniopsis ubonensis 2* | AB698146.1 |
| 15 | *Terniopsis malayana 1* | AB450034.1 |
| 16 | *Terniopsis malayana 2* | AB698132.1 |
| 17 | *Terniopsis microstigma 1* | LC342922.1 |
| 18 | *Terniopsis microstigma 2* | LC342919.1 |
| 19 | *Terniopsi ssessilis* | AB048377.1 |
| 20 | *Tristicha trifaria* | AB113745.1 |
| 21 | *Dalzellia ubonensis* | AB450027.1 |
| 22 | *Dalzellia angustissima* | AB450016.1 |
| 23 | *Dalzellia kailarsenii* | AB450017.1 |
| 24 | *Weddellinas quamulosa* | AB038206.1 |
| 25 | *Polypleurum wallichii* | LC380633.1 |
| 26 | *Zeylanidiumlic henoides* | AB048828.1 |
| 27 | *Tristellateia madagascariensis* | AF344584.1 |

**Table S2 List of taxa from Podostemaceae and NCBI accession numbers** (nrITS)

| 1. No. | Taxa | NCBI accession no. |
| --- | --- | --- |
| 1 | *Terniopsis yongtaiensis* | OM949049.1 |
| 2 | *Terniopsis chanthaburiensis 1* | LC625816.1 |
| 3 | *Terniopsis chanthaburiensis 2* | LC625815.1 |
| 4 | *Terniopsis heterostaminata* | LC625821.1 |
| 5 | *Terniopsis filiformis 1* | LC625817.1 |
| 6 | *Terniopsis filiformis 2* | LC625820.1 |
| 7 | *Terniopsis vapyensis* | LC625826.1 |
| 8 | *Terniopsis ubonensis 1* | LC625825.1 |
| 9 | *Terniopsis ubonensis 2* | LC625824.1 |
| 10 | *Terniopsis malayana* | AB083103.1 |
| 11 | *Terniopsis microstigma* | LC625822.1 |
| 12 | *Terniopsiss avannaketensis* | LC625823.1 |
| 13 | *Cladopus taiensis* | LC380644.1 |
